# WHEN CLOSE TIES LIVE FAR AWAY: PATTERNS AND PREDICTORS OF GEOGRAPHIC NETWORK RANGE AMONG OLDER EUROPEANS

**DOI:** 10.1093/geroni/igz038.2771

**Published:** 2019-11-08

**Authors:** Haosen Sun, Markus H Schafer

**Affiliations:** University of Toronto, Toronto, Ontario, Canada

## Abstract

Using the Survey of Health, Ageing and Retirement in Europe (SHARE, Wave 6 in 2015), this paper examines the structure of older adults’ core discussion networks in terms of their geographical outreach. We also examine how far respondents live from their friends, and how such a connection is conditioned by the presence of a proximate child in the network. Findings suggest that older adults in Northern Europe are more likely to have a confidant at mid- and long-range (5-25km and >25km, respectively) than seniors in Central Europe, while their counterparts from Eastern and Southern Europe are less likely to identify a discussant out of their 5km radius. This pattern persists when focusing only on non-kin members of one’s network. However, having a nearby child confidant does not affect the probability of being connected to friends at variant distances in North Europe, while it does predict a lower likelihood of having close-by (0-5km) and long-distance (>25km) friends in Eastern and Southern regions. Other significant predictors of one’s geographical network reach, such as education, financial standing, cognitive ability, computer skills, and car ownership are also discussed and compared across European regions.

